# Pleural Neoplasms—What Could MRI Change?

**DOI:** 10.3390/cancers15123261

**Published:** 2023-06-20

**Authors:** Michał Szczyrek, Paulina Bitkowska, Marta Jutrzenka, Aneta Szudy-Szczyrek, Anna Drelich-Zbroja, Janusz Milanowski

**Affiliations:** 1 Department of Pneumology, Oncology and Allergology, Medical University of Lublin, 20-090 Lublin, Poland; 2 Collegium Medicum, University of Warmia and Mazury in Olsztyn, Aleja Warszawska 30, 11-041 Olsztyn, Poland; 3 Department of Haematooncology and Bone Marrow Transplantation, Medical University of Lublin, 20-090 Lublin, Poland; aneta.szudy-szczyrek@umlub.pl; 4 Department of Radiology and Neuroradiology, Medical University of Lublin, 20-954 Lublin, Poland

**Keywords:** pleura, pleural imaging, pleural neoplasms, pleural tumors, MRI, chest MRI

## Abstract

**Simple Summary:**

The current imaging method recommended in patients with a suspicion of pleural malignancy is CT which has been shown to have certain downsides and limitations—from requiring the administration of the contrast agent and a relatively high radiation dosage to its restricted capacity for the differentiation of the pleural malignancies from the surrounding tissues. During the last few years, numerous studies have suggested that MRI could provide a solution to some of these issues, as various MRI sequences could not only detect and delineate pleural tumors more effectively than CT but also provide additional data on the tumors’ physiology or histology. In this review we summarize current knowledge on the primary pleural neoplasms and discuss potential applications of MRI in patients with pleural malignancies, as well as the current limitations of both the method itself and the research involving it.

**Abstract:**

The primary pleural neoplasms constitute around 10% of the pleural tumors. The currently recommended method for their imaging is CT which has been shown to have certain limitations. Strong development of the MRI within the last two decades has provided us with a number of sequences that could potentially be superior to CT when it comes to the pleural malignancies’ detection and characterization. This literature review discusses the possible applications of the MRI as a diagnostic tool in patients with pleural neoplasms. Although selected MRI techniques have been shown to have a number of advantages over CT, further research is required in order to confirm the obtained results, broaden our knowledge on the topic, and pinpoint the sequences most optimal for pleural imaging, as well as the best methods for reading and analysis of the obtained data.

## 1. Introduction

Immunohistochemistry and histology are considered the only reliable tools to confirm pleural neoplasm diagnoses [1]. Primary pleural neoplasms constitute around 10% of all pleural neoplasms, and the most common ones within that group are malignant pleural mesothelioma (MPM) and solitary fibrous tumor (SFT) [2]. The cytological examination of pleural fluid and needle-aspiration pleural biopsy has shown poor MPM sensitivity—respectively 26% and 20.7% [3,4,5,6]. For that reason, the diagnosis of MPM is often made with the use of image-guided core biopsy or surgical biopsy [2]. The image-guided core biopsy has a sensitivity of 77–86% (with better results obtained with CT guiding than with ultrasound (US) guiding) [2,3] and a 4% seeding rate [2]. The sensitivity of the surgical biopsy ranges from 94% to 100%; however, this procedure is also associated with a 22% seeding rate [2]. Video-assisted thoracoscopy has a sensitivity of 95% to 98% [6,7]; however, tumor seeding occurs in up to 20% of the patients [5]. Since for the currently used methods, the higher sensitivity is connected with an increased seeding rate, physicians are forced to make trade-offs between the method’s efficiency and the patients’ safety.

The use of CT is cheaper than the use of MRI [8,9,10,11] and its availability is higher than that of the MRI [9,10,12]. However, MRI could be used as an alternative method of pleural imaging in patients with contraindications for the use of iodinated contrast [8,13,14,15]. An important downside of the MRI is the presence of numerous artifacts (susceptibility artifact, aliasing, motion artifact) [1,16,17,18], which can only be limited to a certain degree [1,16]. However, for some patients, it could be the safest or the only safe imaging method. Already in 2003, Eibel et al. described the MRI as the superior method to the CT for imaging of pleural diseases [18]. It has been shown to offer a superior contrast resolution and higher accuracy for detecting the invasion of the diaphragm, mediastinum, and chest wall [15,19]. If proven effective, MRI could be used as a non-invasive tool for pleural tumor detection and characterization, with an increase in patients’ safety and comfort.

## 2. Materials and Methods

A literature review focused on the MR’s role in pleural neoplasm imaging was performed with the use of the following search engines: PubMed, Google Scholar, ScienceDirect. This article does not review other pleural imaging methods fully, but it does refer to them in order to show the advantages as well as the pitfalls of the currently available MRI techniques. Comparisons to CT are drawn as comparisons of the MRI to the current standard for pleural imaging.

We would like to stress that this review is focused on pleural neoplasms and as such it does not include detailed information on the tumor-like conditions of the pleura (also known as non-neoplastic).

## 3. Pleural Neoplasms and Their Classification

Pleural neoplasms are classified into four groups: mesothelial tumors, mesenchymal tumors, lymphoproliferative disorders, and pleural metastases. The mesothelial lesions are further divided into benign and preinvasive tumors, and mesotheliomas [20,21] (Table 1).

### 3.1. Mesothelial Tumours of the Pleura

#### 3.1.1. Benign and Preinvasive Mesothelial Tumors

The adenomatoid tumor (AT) commonly grows in the uterus, the fallopian tubes, and the para-testicular area [22,23]. Pleural ATs are extremely rare, with fewer than five cases reported until 2009 [23].

Well-differentiated papillary mesothelioma (WDPM) usually affects the peritoneum of women of various ages and has a slow-progressing clinical course with long patient survival [24]. WDPM of the pleura is very rare, with less than 100 reported cases [24]. There is no standard treatment for it; however, the use of chemotherapy and radiotherapy has been reported [24].

Malignant mesothelioma is proceeded by an in situ tumor, whose timely identification and removal may prevent its transformation into an invasive form [25]. It is associated with asbestos or radiation exposure, as well as familial predisposition [26]. The patients present non-resolving pleural effusions and show no thoracoscopic or imaging evidence of the tumor [21,23]. After a median follow-up time of 60 months, up to 70% of mesotheliomas in situ will progress into an invasive form [25,27]. The diagnosis of benign and preinvasive mesothelial tumors is mostly based on histopathology, immunohistochemistry (IHC), and FISH [21,23,24,26].

#### 3.1.2. Mesothelioma

Pleural mesotheliomas (MPM) are rare neoplasms with poor prognosis and account for 70–90% of all malignant mesothelioma cases [28]. Most lesions are associated with asbestos exposure (over 20 years-long latency period) [29]. Other risk factors include exposure to erionite, fluoro-edenite, balangeroite, carbon nanotubes, or therapeutic radiation, as well as chronic pleural inflammation and germline mutations [29,30]. Clinical manifestations include cough, dyspnea, chest pain, weight loss, and malaise [31,32]. The median patient survival time is 9–12 months with a 5-year survival rate of 5% [27].

The diagnostic procedures in the case of a patient with suspected mesothelioma and a pleural effusion should begin with thoracentesis and cytological examination of the pleural fluid’s sample [29]. As only one-third of mesotheliomas can be diagnosed this way, a pleural biopsy should be performed as well [29]. Histological tumor subtypes and archeological patterns are essential in staging, management, and prognosis [29,33]. The three major mesothelioma subtypes are: epithelioid, sarcomatoid, and biphasic, which account for 60%, 20–35%, and 10–15% of cases, respectively [31]. The treatment includes chemotherapy, surgery, and radiation therapy [29,34].

Localized pleural mesothelioma (LPM) is a rare solitary tumor, with only around 80 reported cases [35,36]. It occurs in rather young patients. Its connection to asbestos exposure is uncertain [36]. It is associated with a more indolent clinical course with almost 50% of patients cured after surgical resection (median follow-up time of 4.8 years) [36].

### 3.2. Mesenchymal Tumours of the Pleura

#### 3.2.1. Epithelioid Hemangioendothelioma

Epithelioid hemangioendothelioma (EHE) is a rare malignant tumor with an estimated global prevalence below 1:1,000,000 [37,38]. It can involve any tissue but the most commonly affected organs are the liver, lungs, and bones [38,39,40]. Pleural epithelioid hemangioendothelioma (PEH) is an extremely rare EHE subtype, and has a more aggressive clinical course and worse prognosis than its counterparts in other organs [39,41]. According to the so far documented cases, it usually affects elderly men. The most typical clinical features are dyspnea, chest pain, cough, and fever [37,38,41]. It may mimic other diseases, such as tuberculosis, metastatic cancer, or pleural mesothelioma [42,43]. It is commonly misdiagnosed, which contributes to the average survival time of just 10 months and the 5-year survival rate of 5% [37,42,44].

The diagnosis of PEH is mainly based on the histopathological examination of a sample retrieved through lung biopsy or thoracoscopic biopsy [42,44]. There is no set PEH treatment standard [39,44]; however, the use of methods such as pleural decortication, chemotherapy, and immunotherapy has been reported [37].

#### 3.2.2. Angiosarcoma

Angiosarcoma can affect several organs [45,46] and usually appears in the pleura as a metastasis from other tissues [47]. Primary pleural angiosarcoma (PPA) is extremely rare and displays heterogeneous clinical presentation [45,46,47]. The diagnosis is usually set based on the biopsy results [45,48]. The treatment involves surgical resection, radiotherapy, or chemotherapy [45,46,48]. Based on the limited number of reported cases, the 2-year survival rate for patients with PPA seems to be around 4.4% with a median overall survival of 4 months [48].

#### 3.2.3. Synovial Sarcoma

Synovial sarcoma (SS) is a rare mesenchymal neoplasm that can be found in the deep soft tissue of the upper and lower extremities [49,50]. The pleural location of SS is atypical and associated with a more aggressive disease course [51]. Primary pleural synovial sarcoma (PPSS) cases constitute less than 1% of all primary lung malignancies. It usually presents in patients aged 30–50 with unspecific symptoms [49,52]. The diagnosis is made based on the histopathological examination and IHC [50]. Treatment for PPSS is unspecific, but typically includes surgery, chemotherapy, and/or radiotherapy [49,52].

#### 3.2.4. Solitary Fibrous Tumor and Malignant Solitary Fibrous Tumor

Solitary fibrous tumors (SFTs) of the pleura are uncommon and usually benign [53,54]. They typically affect people in their sixth decade of life and have an asymptomatic beginning, causing more symptoms as they grow [54]. On rare occasions, children might be affected as well [55]. The diagnosis is based on histopathological examination and IHC [53,54,56]. The preferred treatment method is surgical resection [53].

#### 3.2.5. Desmoid-Type Fibromatosis

Desmoid-type fibromatoses or desmoid tumors are locally aggressive but non-metastatic neoplasms with an unpredictable clinical course and a propensity for local recurrence [20,21,57]. Primary pleural desmoid tumors are extremely rare [57,58]. Their diagnosis is based on the biopsy results [20]. The treatment might include patient observation, surgery, radiation therapy, chemotherapy, or administration of hormonal agents [59].

#### 3.2.6. Calcifying Fibrous Tumor

The calcifying fibrous tumor (CFT) is a benign lesion occurring in various parts of the body [60,61]. Around one-tenth of CFTs are pleura-based; however, it is unclear whether they occur there as a result of spreading from different organs or as one of many primary tumor foci developing simultaneously [60].

Calcifying fibrous tumor of the pleura (CFTP) often has an asymptomatic course [61] and occurs more frequently in adult women [56]. The diagnosis is based on histopathological examination and IHC [56,61,62]. Surgical resection is considered to be the best treatment method [60].

#### 3.2.7. Desmoplastic Round Cell Tumor

Desmoplastic small round cell tumor (DSRCT) is characterized by aggressive behavior and poor prognosis with a 5-year survival rate of 15–30% [63,64]. It has an incidence of approximately 0.2 to 0.5 cases per 1 million [65] and usually arises in the abdomen. DSRCT of pleura is extremely uncommon and can manifest with chest pain, pleural effusion, or dyspnea [56,63,66]. Men are affected approximately four to five times more often than women [65,67]. Histopathological examination and genetic testing are useful in the diagnostic process [56,63]. So far, a universal treatment protocol has not been established; however, it usually includes a combination of surgery, radiotherapy, and chemotherapy [63,66].

### 3.3. Lymphoproliferative Disorders of the Pleura

#### 3.3.1. Primary Effusion Lymphoma

Primary effusion lymphoma (PEL) is an uncommon non-Hodgkin lymphoma that is localized predominantly in serous body cavities such as the pleural cavity. It is associated with the human herpes virus type-8 infection (60–90% of cases) and a possible concomitant EBV infection in immunocompromised individuals [56,68,69,70]. Patients with PEL present serous effusions which may create a mass effect, with the absence of lymphadenopathy, organomegaly, or solid masses [56,68,69,70]. Primary diagnosis is made based on the analysis of the effusion’s content and confirmed with the detection of the HHV8 infection in the nuclei of the cancer cells. The treatment includes chemotherapy and anti-retroviral therapy. The median survival period for patients with PEL is less than 6 months [69,70].

#### 3.3.2. Diffuse Large B-Cell Lymphoma Associated with Chronic Inflammation

Diffuse large B-cell lymphoma (DLBCL) is an EBV-associated lymphoma that presents itself as a mass, most commonly located inside the pleural cavity, where it develops after a long history of pyothorax (20–64 years). The patients tend to report chest or back pain and fever [56,71,72,73]. Pleural DLBCL is aggressive with a median patient survival of less than 12 months and a 5-year survival rate of around 21%. A wide range of IHC stains may be used to support the diagnosis. The treatment methods include surgical resection, radiotherapy and/or chemotherapy [56,72,73].

### 3.4. Pleural Metastases

Metastases are the most common neoplasms found in the pleura [39] and may derive from a variety of tumors [11,74,75,76,77].

## 4. MRI as a Diagnostic Tool in Pleural Neoplasms

### 4.1. MRI and Its Potential as a Diagnostic Tool in Pleural Malignancies

To this day, contrast-enhanced CT is the first-choice imaging method in patients with a suspected pleural malignancy [1,4,8,10,14,16,17,28,75,78,79,80,81]. However, in the twenty-first century, a strong development of imagery and postprocessing tools has occurred which has led to an increase in MRI’s diagnostic value [4,14]. It has since been demonstrated that the MRI could complement the CT findings in patients with pleural neoplasms [1,2,12,18,28,82,83,84,85,86]. It could be used to identify and assess the invasion of the surrounding tissues—chest wall, diaphragm, bones, and endothoracic fascia [1,2,15,16,28,82,85,86,87,88,89,90,91,92,93,94]—and provide additional functional information [9]. When it comes to the detection of pleural malignancies and the differentiation between benign and malignant pleural disease, MRI was shown to have sensitivity and specificity values at the very least equal—and in many cases higher—to those of the CT [1,2,4,6,12,75,82,86,95]. MRI could be a radiation-free alternative to CT [80], which makes it more appropriate for repetitive use during follow-up examinations, including in pediatric patients [9].

We have observed a consensus on MRI’s contrast resolution being superior to that of the CT [9,11,15,75,82,96]. However, the results regarding the methods’ spatial resolution seemed to be contradicting, with some sources describing MRI’s spatial resolution as inferior and some as similar to that of the CT [9,11,15,17,75,82,96]. Furthermore, according to a review by Pessoa et al., which was published in 2016, MRI had a better spatial resolution than CT [76].

Currently, the basic MRI sequences recommended for pleural imaging include multiplanar ECG-triggered T1 (T1-weighted), T2 (T2-weighted), and CE T1 (contrast-enhanced T1-weighted) with respiratory triggering and breath-holding techniques [14]. Additionally, some sources suggest the use of fat-saturated T1 MRI [8,17]. T1 images provide a contrast between the pleural abnormalities and the extra-pleural fat [74], while T2 images can be used to obtain tissue-specific data [74]. PDW (proton density-weighted) MRI and T2 MRI were found to be useful for differentiating between the malignant and benign pleural conditions [8]. It has been demonstrated that high-intensity T2 images allowed for the differentiation between the malignant and benign lesions, with all malignant lesions showing high signal intensity [13]. The method had a sensitivity of 100%, specificity of 87%, and negative predictive value of 100% [13].

### 4.2. MRI and Primary Pleural Tumours

#### 4.2.1. MRI and MPM

In 60% of the MPM cases, the tumor presents itself on the right side of the body and in 10% bilaterally [1]. When it comes to imaging, MPM often manifests with pleural thickening, pleural effusions, nodules, and focal masses, with an ipsilateral volume loss [1,2,3,6,11,13,75,77,78,79,97,98]. Fissures’ involvement may be visible in the MRI as well [13]. The pleural thickening may be accompanied by bilateral, calcified, or non-calcified pleural plaques [1,2], which are usually a result of asbestos exposure [8,91,99,100] and are the most common symptom of asbestos-related disease [101]. The MPM-related pleural thickening usually presents itself as a rind-like pleural involvement [75]. The nodular mass-like thickening is more specific to the tumor than the smooth thickening, which can be caused by other factors (e.g., an infection) [75,78]. In its more advanced stadiums, the disease may present itself with intra-thoracic and extra-thoracic lymphadenopathy or with metastases [1,2] to the bones [1], liver, spleen, thyroid [78], and brain [1,78]. Since the MPM is strongly associated with asbestos exposure [1,2,3,6,10,18,75,76,78,79,82,83,84,85,90,92,97,98,99,102,103,104,105,106,107,108,109], the imaging might also include other changes typical for asbestos-related diseases [2,78]. In a study by Weber et al. MRI had higher inter-observer agreement values than CT in terms of the pleural asbestos-related pathologies’ assessment [91].

In 1995 the International Mesothelioma Interest Group (IMIG) and the International Association for the Study of Lung Cancer (IASCL) proposed an international MPM TNM staging system [13,110] which was primarily based on surgical and pathological findings, but could be applied to CT and MRI [13]. This meant it could be used to run more accurate treatment selection [13]. The said staging system proposed new descriptors for the T status, while those for the N and M status remained identical to the descriptors used in the International Lung Cancer Staging System [13]. The system did not fully reflect the complexity of the pleural drainage system in its N classification, which was constructed based on the lung cancer one and has since been updated [106,110]. In a TNM 8 staging system, the nodal staging is more aligned with the patterns of the pleural drainage [75]. Proper assessment of the MPM’s stage is important for management decisions [111].

In CT images mesothelioma’s tissue attenuation is similar to that of the adjacent structures (chest wall muscles, diaphragm, pericardium) or complicated pleural effusions [49,60]. Compared to CT, MRI offers superior tissue contrast [88,112,113] which makes it a better tool for the evaluation of the chest wall, diaphragm, mediastinum, and endothoracic fascia in patients with MPM [3,10,75,85,88,91,92,111]. Moreover, MRI provides higher contrast between the tumor and the adjacent effusion [88]. MRI is not routinely performed in MPM patients due to its high cost, relatively low availability, and long imaging time [104]; however, in certain MPM cases, MRI could be used to obtain additional information that may not be available in CT [82,92,113]. Currently, MRI is recommended for MPM imaging in patients in which the detection of T4 stage features could affect the treatment decisions [3,85,88,106]. It has been shown that the ability of the MRI to assess the MPM resectability was higher than that of the CT, in the case of the diaphragm and pleura—most likely due to the additional coronal and sagittal images [14,90,104]. Botticella et al. found that MRI could be used for more accurate tumor delineation and reduce the risk of geographic miss [114].

DCE MRI is used for functional imaging [107,115]. It tracks the signal changes after a passage of an administered contrast agent through rapid sequential imaging [10,108]. DCE could be used to assess the vascularity, perfusion, and vascular permeability of the pleural malignancies and predict their response to chemotherapy [1,11,16,17,74,75,82,107,108,112,115,116,117]. A two-compartment mathematical model may be used for pharmacokinetic analysis, to quantify the changes in microcirculation and judge vascular permeability [10,82].

The AUC (area under the curve) reflects the gadolinium’s behavior of synchronous flow, as well as the permeability and compartmental volumes of the tumor [107,116]. It is relatively robust and does not require model fitting [116]. Tomšič et al. found that the AUC values were higher in patients who obtained disease control after receiving cisplatin-based chemotherapy than in patients who experienced disease progression [116].

Ktrans is the volume-transfer constant between the plasmatic and extravascular extracellular space, calculated based on the DCE images [10,107]. Ktrans values are primarily related to either vessel permeability or blood flow [107,116]. A decrease in Ktrans values in MPM could reflect a decreased vessel permeability which is expected as a result of the leaky neovessels’ normalization which was shown to be a sign of a present tumor response [116]. However, when focused primarily on blood flow, increased Ktrans values could point toward improved drug delivery and be a positive prognostic factor—as has been observed in other tumor types [116]. Higher pre-treatment Ktrans values were found to predict a better treatment response and longer survival time of the MPM patients receiving chemotherapy, since more permeable vessels meant a better drug delivery to the MPM cells [107]. Additionally, Ktrans could provide information on the tissue’s oxygenation and thus be a prognostic factor in patients undergoing radiotherapy [107].

Giesel et al. [115] found that the Amp value obtained in the pharmacokinetic analysis of the DCE MRI could provide an insight into tumor’s vascularity, as it is directly related to the tumor vessels’ permeability [115].

Ve describes the extracellular extravascular volume measured in the DCE MRI [10] and its increase could reflect cell death [116].

Kep is the rate constant in the DCE MRI [10]. Numerous studies found a correlation between the pre-treatment and inter-treatment Kep values, their changes, and the clinical outcome. However, we observed a disagreement as to what the specific changes meant [10,107,116]. Further research in this direction is required to clarify the Kep’s significance.

Tsim et al. [42] observed a good performance of the ECE (early contrast enhancement) in determining the malignant pleural lesions from the benign ones, which could make it a better tool for pleural malignancies’ detection in its early stages [42]. It has also been found that the CE MRI-measured tumor volume was a better predictor of the patients’ overall survival than the CE CT-measured tumor volume, clinical T-stage, and overall disease stage. It was also the only imaging-based independent prognostic factor for MPM, when dichotomized at 300 cm^3^ [88]. The superiority of the CE MRI-based volumetry over the CE CT-based volumetry could be explained by a manual tumor segmentation that promotes reader-related inaccuracy in the case of the CT analysis [88]. Additionally, CE fat-suppressed sequences were found to be the most sensitive to the infiltration of the interlobular fissures and adjacent tissues [14,75,104]. However, the DCE MRI’s spatial coverage is often limited [10].

**Figure 1:**
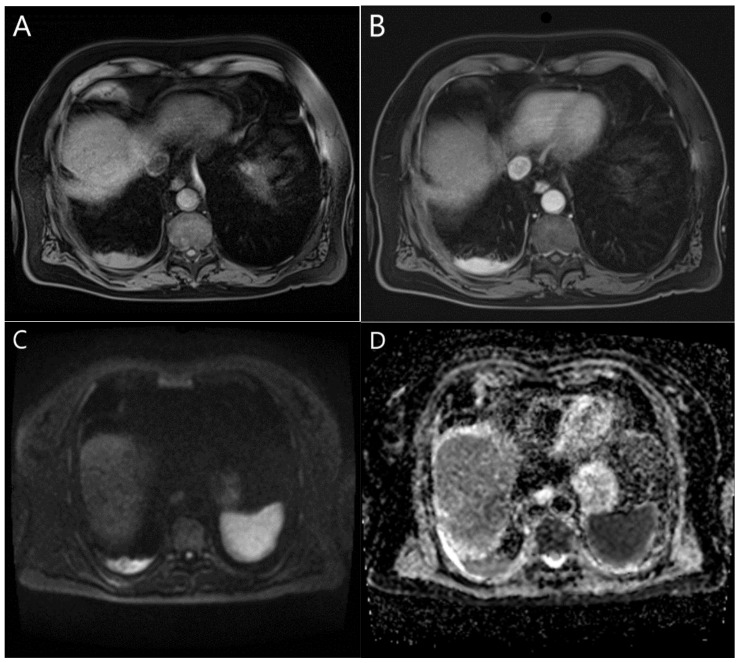
MR images indicate thickening/consolidation within the dorsal aspect of right pleural cavity, consistent with mesothelioma. Pre- (**A**) and post-contrast (**B**) T1-weighted vibe axial images with fat saturation depict vividly enhancing lesion, with apparent signs of diffusion restriction on high b-value (b = 800) DWI images (**C**) and complimentary ADC maps (**D**).

The diffusion-weighted MRI (DW MRI) is based on the differences in the tissues’ water mobility [4,5,10,118]. It provides information on their cellularity, perfusion, disorganization, and extracellular space [5,14,104], and could be at least as efficient as FDG-PET or PET/CT in differentiating between benign and malignant pleural lesions [4,10,118,119]. Tissues that strongly limit the water molecules’ movement—as in mesothelioma—usually display higher signal intensity than normal tissues or areas with free water movement—such as effusions [10] (Figure 1). Jiang et al. reported that DWI could be applied in patients with a suspicion of pleural malignancy in order to avoid unnecessary invasive procedures, and to judge the presence of the N3 lymph nodes, as well as the extra-thoracic metastases [4]. This could be of large importance for MPM staging since the presence of an N3 lymph node or of a distant metastasis is considered a contraindication for the resection [85].

Water mobility can be quantified by calculating the apparent diffusion coefficient (ADC) [10], which could be used to differentiate between benign and malignant lesions [10,14]. It has been reported that the epithelioid subtype had higher ADC values than the sarcomatoid type [2,10] and biphasic type [10]. Additionally, ADC could be used to monitor the patients’ treatment response. The tumor density lowers in the case of a successful treatment, which allows higher water molecules’ mobility and leads to an increase in the ADC values [10]. However, despite very promising results regarding DW MRI and ADC, some overlap in the ADC’s values in malignant and benign lesions has been reported [4,120], which so far made it impossible to establish a sufficiently precise cut-off value [120]. Another downside of the ADC is that it depends on subjective radiological evaluation [4].

In PDW (proton density-weighted) MRI, the pleural thickening, nodules, and masses occurring in MPM were reported to produce a hyperintense signal in relation to the chest wall muscle [1,2,92].

Ohno et al. found that the whole-body MRI and FDG-PET/MRI with signal intensity (SI) assessment were the most accurate imaging methods for the MPM’s TNM evaluation, and could replace FDG-PET/CT. The researchers recommended using whole-body MRI and FDG-PET/MRI over the whole-body CT for the ISACL mesothelioma staging [103].

In research on MPM patients, Murphy et al. found FDG-PET/MRI to be more accurate in tumor staging than FDG-PET/CT, due to its better soft tissue resolution. However, FDG-PET/MRI and FDG-PET/CT were shown to be similarly effective as staging tools [87]. Diffusion restriction areas observed in MRI were consistent with the increased FDG uptake in patients with MPM [87].

In 2022, Volpi et al. proposed an MRI protocol for MPM. The authors recommended the 2D T2-weighted acquisition on an axial, coronal, and sagittal plane, and a T1-weighted turbo spin echo (TSE) sequence for the morphological analysis, an axial DWI with multiple b-value (b = 0, 50, 400, 800 s/mm^2^) for functional imaging, and T1-weighted fat-saturated 3D gradient echo (VIBE) sequences before and after contrast injection to assess the lesions’ enhancement [121]. Romei et al. [113] recommended applying the mRECIST to MRI, in order to assess the tumor’s early response to treatment.

#### 4.2.2. MRI and SFT

Solitary fibrous tumors, otherwise known as localized fibrous tumors make up 5–10% of the pleural neoplasms [2,54,74,75,77,79,122]. Out of those, approximately 12–40% are malignant [2,13,55,122,123,124,125]. The majority of the pleural solitary fibrous tumors (65–80%) originate from visceral pleura [2,75,122,123,125]. They typically occur as single lesions; however, conglomerate or multifocal masses could be observed in rare cases [123]. An SFT usually presents itself as a well-defined, smooth, or lobulated homogenous mass, adjacent to the diaphragm and encapsulated in a serous membrane [75,123,124,125]. Around 40–50% of the solitary fibrous tumors have a vascular pedicle that attaches them to the pleural surface [75,122,123]. Calcifications are rare and more common in larger tumors [79,122]. Accompanying pleural effusions are not common either [122]. The tumor may adhere to the adjacent pleural surfaces or to the pericardium [123]. Even though SFTs are often encapsulated, they can still be focally invasive [122]. It has been observed that benign solitary fibrous tumors transform into malignant lesions after several years [123], which could be related to the p53 mutation [55]. Some SFTs may mimic diaphragmatic eventration [75].

In MRI SFTs’ characteristics resemble those of fibrous tissue [75]. It has been shown that MRI could detect the presence of the intra-tumoral flow void within an SFT [122]. It could also be superior to CT in delineating larger solitary fibrous tumors from the adjacent tissues and used to confirm the tumor’s localization in the case of a diaphragm-abutting tumor [123,124]. T2 MRI could be helpful in determining the benign SFTs from the malignant SFTs—the latter are likely to be hyperintense due to high vascularity, cellularity, and edema [124]. This feature could be especially useful, as it has been reported that not only malignant SFTs but also around 60% of the benign SFTs were heterogeneously enhanced in the CT images, which makes CT a poor tool for differentiating between them [122]. The coronal and sagittal images in MRI facilitate the diaphragmatic evaluation in patients with solitary fibrous tumors [123].

In DW MRI the SFTs have been reported to have a low or intermediate signal intensity due to the fibrous tissue’s presence [75,79,123].

After SFT’s resection CE CT follow-ups are recommended twice a year for the first two years and once a year afterward [125]. MRI could be a way to limit the patients’ exposition to radiation.

#### 4.2.3. MRI and Lymphomas

Pleural involvement is observed in up to 30% of all lymphoma cases [11]. Pleural lymphomas usually occur due to recurrence or an extension of the disease [13]. Such secondary pleural lymphomas can be observed in up to 20% of the patients [76,126]. Primary pleural lymphomas constitute around 7% of primary lymphoma cases and are usually observed in patients with chronic diseases of the pleura [2,76]. Primary pleural effusion lymphoma is a herpesvirus-8 positive DLBCL (diffuse large B-cell lymphoma) [85] which can usually be observed in patients infected with HIV [126,127]. In imaging, the pleural effusion is a main finding, and no solid tumor is observed [126,127]. Pyothorax-associated lymphoma (PAL) is an Ebstein–Barr virus positive DLBCL [127]. It is associated with a chronic inflammatory pyothorax and presents itself as a solid tumor that may be accompanied by a pleural effusion [127].

Lymphomas in MRI can take the form of solitary nodules or multiple broad-based pleural plaques [13]. Around 20–30% of the lymphoma patients have pleural effusions [126,127]. MRI was shown to be superior to CT in detecting and evaluating chest wall invasion in lymphoma patients. It was also more effective than CT in recognizing the invasion of the pleura [13]. CE MRI could also differentiate between the lymphoma and a similarly presenting empyema, since the signal from the latter does not enhance [2]. Pleura’s assessment is especially important for proper staging and treatment strategy in the case of Hodgkin lymphoma [11].

#### 4.2.4. MRI and Pleural Lipoma and Liposarcoma

Pleural lipomas are rare [75,128] and could be diagnosed with the use of both CT and MRI [8,128]. In MRI a well-defined homogenous mass can be observed [74]. The tumor may be surrounded by a pseudo-capsule [11]. Lipomas are hyperintense in T1 images and moderately intense in T2 images [74]. In the case of diagnostic doubts fat-suppression sequences can be used [74,75]. In comparison, liposarcomas show incomplete suppression in fat-saturated sequences [74]. In MRI liposarcoma may present itself as a heterogenous mass, as it does contain a mixture of fat and soft tissue which vary in density [77].

#### 4.2.5. MRI and Pleural Leiomyoma and Leiomyosarcoma

In 2017 Haratake et al. reported a case of a pleural leiomyoma [129]. In MRI the tumor presented itself as a well-defined heterogeneous mass similar to the SFT. In this particular case, the authors found the MRI to be helpful in assessing the tumor’s invasion of the surrounding tissues and establishing its resectability [129]. In 2005 Al-Daraji et al. described a case of pleural leiomyosarcoma [130]. The tumor had high vascularity, and contained adipose tissue, as well as fibrotic and myxoid regions [130]. MRI examination was carried out to complete the CT findings and revealed that the tumor was pleura-based [130].

#### 4.2.6. MRI and Other Sarcomas of the Pleura

Pleuropulmonary synovial sarcoma can originate in numerous organs, including the pleura [131]. MRI could provide high-quality images of the nodular soft tissue and the multi-locular fluid-filled internal components of the tumor [131,132]. In CE MRI a peripheral rim enhancement may be observed [131,132]. The tumor may often be accompanied by a pleural effusion. Tumors with focal necrosis, hemorrhage zones, or cysts have been reported as well [132]. Compared to soft-tissue synovial sarcoma, pleuropulmonary synovial sarcoma has lower vascularity [131]. MRI could be used for an accurate tumor delineation from the surrounding tissues [131,132]. A “triple sign” may be observed [131].

Pleural low-grade fibromyxoid sarcoma (PLGFS) is a rare tumor that may resemble mesothelioma in CT images. Despite a somewhat benign appearance, the tumor has a tendency for metastases and recurrence. Typically, PLGFSs are hypo- or isointense in relation to muscle in T1 MRI and show heterogenous high-intensity signals in T2 MRI. Hemorrhages may occur occasionally [133]. Liang and Xu reported on a case of a PLGFS patient. The MRI revealed a cystic solid tumor with a hypointense central part and mildly hyperintense edges in T1 images, high intensity in T2 images, and ring-like enhancement in delayed post-contrast images. The edges’ hyperintensity in T1 was most likely a reflection of the necrosis [133].

Extra-skeletal osteosarcomas are extremely rare, malignant tumors. In 2008 Matono et al. reported a case of pleural osteosarcoma. CT revealed a pleural mass accompanied by a pleural effusion, encasing the left lower lung. MRI showed that the tumor was adjacent to the pleura [134]. A differential diagnosis for these tumors should include MPM [134].

#### 4.2.7. MRI and Pleural Hemangioma

Pleural hemangiomas are extremely rare [135,136,137]. According to Yoldi et al. there had only been two cases of pleural hemangioma reported until 2016, out of which one was accompanied by a pleural effusion [137]. While the CT enables the evaluation of the tumor’s location, morphology, and extent, MRI provides detailed information on the tumor’s extent and its tissue, as well allowing it to differentiate the tumor tissue from the adjacent inflamed tissues [135]. Pleural hemangiomas should be considered as one of the differential diagnoses in patients with recurrent unilateral pleural effusions [138].

#### 4.2.8. MRI and Pleural Hemangioendothelioma

Pleural epithelioid hemangioendotheliomas (PEHs) are extremely rare [41]. In 2000 Crotty et al. retrospectively examined the data of four patients with pathologically confirmed PEH [139]. The researchers analyzed the four patients’ CT scans and the MRI results of one patient, who was the only one to receive said imaging [139]. They reported that in all four patients, the tumors were located on the right side of the chest [139]. In the CT scans both smooth and nodular pleural thickening was observed in all patients. Multiple pulmonary nodules were noted in two patients [139]. In MRI PEH presented itself with pleural thickening, nodularity, and a loculated pleural effusion [139]. In 2008 Lee et al. described a case of a patient with PEH. The tumor presented itself as a nodular pleural thickening with an extra-pleural tumorous lesion and multiple subpleural nodular lesions, and was located on the right side of the chest [41]. Vertebral metastases were identified [41]. All of the above data were obtained from CT scans [41]. The authors did not report the use of MRI in the case of this patient [41]. In 2021 Askari et al. described a case of a patient with PEH on the right side of the chest. In imaging pleural effusion and pleural thickening were observed. No MRI use was reported in this case either [140].

PEH needs to be differentiated from pulmonary EH (pulmonary epithelioid hemangioendothelioma) because the tumors have different prognoses and treatments [41]. PEH is more aggressive than other EH subtypes [140] and the prognosis in the case of PEH is generally much worse than for pulmonary EH [139]. The sex and age of the patients could potentially point the physicians in the right direction, as the PEH is more often observed in older men, while the pulmonary EH is primarily observed in young and middle-aged women [46,47,48]. Exposure to radiation or to asbestos is considered a risk factor and its confirmation could be helpful in establishing the diagnosis [140]. Crotty et al. reported that in radiographs PEH could resemble mesothelioma or diffuse pleural carcinomatosis [47], which should be included in the differential diagnosis, together with metastatic pleural disease, sarcomas, pseudo-pyogenic granuloma, tuberculosis, and hyperplasia [139,140].

#### 4.2.9. MRI and the Peripheral Nerve Sheath Tumors

Peripheral nerve sheath tumors include neurofibromas and Schwannomas [141]. Although the mediastinal involvement of neurofibromas is fairly common, pleural involvement is rare [141,142]. In 2009 Rathinam et al. described two cases of pleural neurofibromas; however, no MRI use was reported in these cases [142]. In 2021 Sharma et al. [141] reported a case of a primary pleural neurofibroma. The tumor was hypointense in T1 images and heterogeneously hyperintense in T2 images [141]. The differential diagnosis should include SFT [142].

Schwannomas are usually benign, slowly growing tumors [143,144,145]. Pleural Schwannoma’s occurrence is extremely rare [143,144,146] and is usually located in the posterior mediastinum [144]. In 2016 Ochtrop et al. described a case of a pleural psammomatous Schwannoma in a 33 years-old patient [143]. In T2 MRI hyperintense liquid parts of the tumor could be observed, while the CE T1 MRI revealed an inhomogeneous uptake of the contrast agent [143]. The mass had septations, high vascularity, and smooth edges [143]. In 2017 Bibby et al. described a case of an encapsulated pleural Schwannoma with multiple cysts on its surface [145] However, Schwannomas could also present themselves without the characteristic well-defined borders [144]. Benign pleural Schwannomas should be distinguished from malignant pleural Schwannomas in which pleural nodules, pleural effusions, or pulmonary metastases may occur [143]. Since the imaging results are not tumor-specific, a histological investigation is required to set the diagnosis [143]. Pleural effusions can often accompany malignant Schwannomas, but are uncommon for benign tumors which occur around 11 times more frequently than malignant ones [145].

#### 4.2.10. MRI and DSRCT

Desmoplastic small round cell tumors (DSRCTs) usually (around 90–93% of cases) originate from the peritoneum [65,67,147]. However, in rare cases, they may also originate from the pleura. Pleural DSRCTs are mainly seen in young adults; however, in 2006 Karavitakis et al. described a case of a 10-year-old with a pleural DSCRT, which presented itself as a solid paraspinal tumor in MRI [147]. Pleural nodules that may be observed in patients with DSRCT [147] are typically hypointense or isointense in comparison to muscle in T1 images and heterogeneously hyperintense in T2 images [67]. Pleural effusions were reported rarely in patients with pleural DSRCT [65].

Table 2 gives an overview of the pleural tumors’ presentation in MRI sequences.

### 4.3. MRI and Pleural Infiltration and Metastases

#### 4.3.1. MRI and Thymoma

Thymomas show a tendency for pleural extension and seeding [13,74,75]. Post-surgery recurrence of the disease in the form of pleural seeding can be observed often as well [13]. In T1 thymoma looks isointense to skeletal muscle and in T2 images it gives a signal intensity similar to that of fat. CT or MRI is recommended for the follow-up examination of thymoma patients [13].

#### 4.3.2. MRI and Bronchial and Lung Cancer

Lung tumors are responsible for around 36% of malignant pleural effusions [13]. MRI was shown to be better than CT at discerning soft tissue and vascular planes in lung cancer patients [7], and could be used to assess pleural involvement [7,148]. Zhang et al. demonstrated that despite numerous similarities in terms of efficiency, the CE radial T1 gradient-echo 3T (Teslas) MRI was better than CT in detecting the visceral pleura’s invasion in patients with non-small cell lung cancer (NSCLC) [149].

The most common cause of pleural malignancy is bronchogenic carcinoma [11,13,74,75,76,77]. MRI is a better tool than CT for evaluating the infiltration of the parietal pleura and chest wall by bronchogenic carcinoma [13]. A tumor infiltrating the visceral pleura is classified as a T2 lesion, one extending into mediastinal or parietal pleura as a T3 lesion, and one with a cytologically malignant pleural effusion—as an unresectable T4 [13]. A benign reactive pleural effusion is insignificant for the staging of bronchogenic carcinoma [13].

#### 4.3.3. MRI and Pleural Metastases

**Figure 2:**
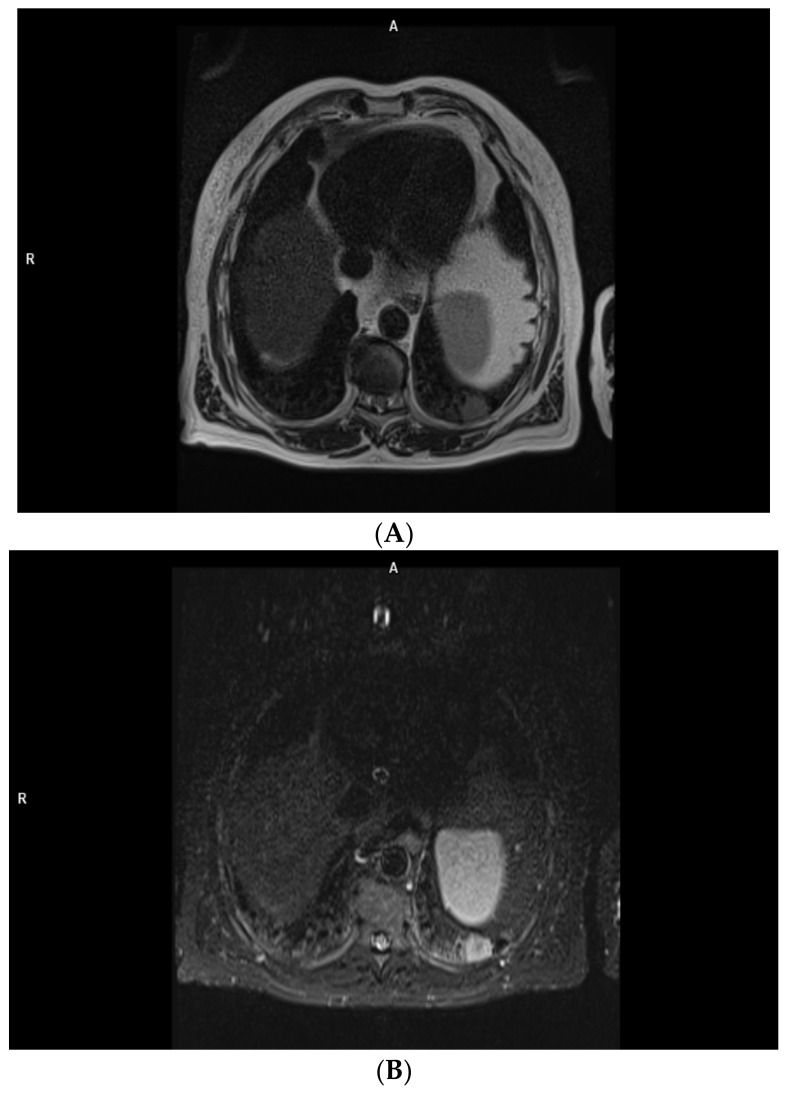

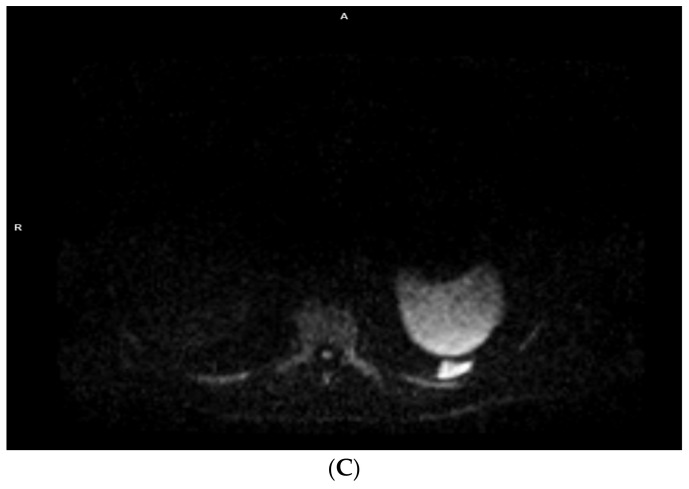
Images of a 70-year-old male patient with colorectal cancer. Focal lesion (meta) adjacent to the dorsal pleura at the base of the left lung. T2-weighted (**A**), TIRM (**B**), and DWI b = 800 (**C**) axial images.

Metastases’ presentation in MRI is dependent on the MRI presentation of the primary tumor [76]. The unilateral metastatic pleural disease can be hard to distinguish from MPM [74]. Imaging may show circumferential or lobulated pleural thickening, pleural effusions, rib crowding, and elevated hemidiaphragm [19]. If the ADC of the primary tumor is known, it can be used as a marker for metastases’ detection in DW MRI [76]. The most common cause of malignant pleural thickening is pleural metastases, of which 40% derive from bronchogenic carcinoma and 20% from breast cancer (followed by lymphoma, ovarian cancer, and gastric cancer) [11,74,75,76,77]. Pleural effusions are the most common finding in patients with pleural metastases [13] or MPM [98]. MRI detects pleural effusions at least as effectively as CT [150]. They typically have low signal intensity in T1 and high signal intensity in T2 imaging [75,79,92] (Figure 2).

### 4.4. MRI and Selected Pleural Pathologies That May Be Associated with Pleural Neoplasms

In comparison to CT, MRI has a superior soft tissue contrast resolution [10,11,18,19,74,76,82,96,104,126,151]. It was shown to outperform the CT when it comes to detecting diffuse pleural thickening, malignant pleural thickening, extra-pleural fat, and pleural effusions [75,79]. It was also better than the ultrasound examination (USG) at detecting and characterizing pleural effusions [76]. It has been shown that the malignant pleural thickening was hyperintense [74] and benign pleural thickening was hypointense [75] in relation to intercostal muscle. Sagittal and axial T2 sequences could be especially useful for detecting nodular pleural thickening [79]. The excellent contrast resolution of the MRI can be further enhanced through the administration of contrast agents [74,82,104]. Fat-saturated CE MRI could be used to detect more subtle malignant pleural thickening [74,79].

In the absence of contrast, fluid (e.g., from pleural effusion) and extra-pleural fat display high signal intensity in T2 MRI [17], which contrasts with the low-intensity signal produced by the parietal pleura. This often allows the recognition of the pleural nodularity without the use of contrast agents [75,79]. For the same reason, MRI is a good tool for detecting septations, which may be hard to detect in CT [74,75,79].

The triple echo (TE) pulse sequence, as well as DW MRI (ADC values), could differentiate between the exudate and transudate pleural fluid which could help identify the cause of pleural effusion [74,79,119]. Contrast enhancement and the T2 relaxation time measurement could be used to distinguish the exudate from the transudate as well [11] (Table 3).

Malignancy should be suspected if the pleural effusion is accompanied by circumferential pleural thickening, mediastinal pleural involvement, nodularity, or is more than one centimeter thick [11,152]. Malignant pleural effusion can be confirmed after the detection of the neoplastic cells in the pleural space [12].

Pleural plaques on their own are benign changes without risk of malignant transformation and usually do not cause functional impairment [101]. However, they can also be related to pleural malignancies [75,101]. In MRI pleural plaques show low signal intensity in T1, T2, and PD sequences [75,79,101] and are minimally enhanced after the administration of a gadolinium contrast agent [75]. In one study the pleural plaques were isointense to muscle in T1 images [91]. Although MRI was shown to detect the pleural plaques almost as well as CT, the latter has been deemed better for detecting calcification and remains the golden standard for plaque identification and characterization [8,17,74,75,79,91,153].

Coolen et al. found that the detection of pleural pointillism in the MRI reached an 88% accuracy in detecting malignant pleural disease [154].

Parallel MRI acquisition methods—GRAPPA (generalized auto-calibrating partially parallel acquisition), TrueFISP (fast imaging with steady precession), and FLASH (fast low angle shot)—could be used to identify a subtle spread of the tumor on the neighboring structures (mediastinum, myocardium, vascular structures) [1].

Bone involvement could be detected with the use of STIR (short tau inversion recovery) MRI sequences [8,14]. STIR could also be applied for pleural tumor extension’s evaluation [14].

## 5. Discussion

Based on the so far available data, MRI seems to have the edge over CT when it comes to the assessment of the tumor’s extent in the case of MPM, SFT, and lymphomas [13,88]. MPM and SFT are considered to be the most common primary pleural neoplasms [2]. The risk of acquiring MPM due to asbestos exposure is estimated to be around 10% in the exposed population [11]. So far, we lack tools for MPM screening [106] which could be especially useful in populations with relatively large chances of repeated asbestos exposure. For example, in Mexico where the use of asbestos is still allowed, the MPM incidence is expected to keep rising within the next decades [6]. A screening program for the workers who come in contact with asbestos is highly needed [6]. If proven effective, MRI could provide the solution to the problem above. However, it has been reported that, due to the low prevalence of the MPM, institutional cooperation would be required for a wider study that could confirm the so far obtained results [88].

MRI’s use has also been documented in patients with pleural leiomyoma, leiomyosarcoma, pleuropulmonary synovial sarcoma, PLGFS, and pleural osteosarcoma, in which it was used to obtain more exact tumor characterization [129,130,131,132,133,134]; however, these were only single cases.

When it comes to the pleural lipomas, we did come across the information that both the CT and the MRI were fit to be used for their imaging [8,128]. In 2004 Bittner [11] wrote that CT was sufficient for the diagnosis of lipomas [11]; however, fat-suppression MRI was the recommended method in the case of a diagnostic uncertainty [74,75]. It could also aid physicians in differentiating liposarcoma from lipoma, since only the latter shows complete fat suppression [74].

Outside of the more precise assessment of the tumor’s extent, MRI could be useful for establishing or confirming the character of a pleural lesion in a non-invasive manner. It has been demonstrated that the CE MRI could determine the pleural lymphoma from pleural empyema based on the differences in signal enhancement [2]. The DCE MRI could also be applied to obtain additional information about the tumor’s vascularity and help predict the patient’s response to the treatment [16,17,116].

In the case of fluid accumulation, DWI could be used to establish the underlying cause based on the ADC values [74,79]. Additionally, the DWI results (ADC value) could provide valuable prognostic data in patients with MPM [2] since the epithelioid subtype of the MPM is related to a better prognosis [5,10,13,26] than the sarcomatoid and biphasic subtype [1,2,26]. In a paper published in 2020, Usuda et al. demonstrated that the ADC values of the MPM and pleural lung cancer disseminations could allow their differentiation from benign lesions [118]. Earlier, in 2006, West et al. stated that neither CT nor MRI were able to reliably determine the MPM from the metastatic pleural lesions [90], which would partially align with the results obtained by Usuda et al.

Based on the currently available data, it would seem that the DW and DCE MRI sequences in particular have the potential to determine the character of a pleural lesion and could be used as diagnostic tools in patients with pleural disease. We stipulate that the more challenging part could be the exact determination of the lesion’s type which may be necessary for treatment planning. In particular, patients with unresectable tumors could profit from the introduction of non-invasive diagnostic methods. However, in the absence of an invasive sampling procedure, the MRI would have to make up for it and precisely determine the tumors histopathological profile. Although promising, the MRI’s potential to tell the benign and the malignant lesions apart needs to be further investigated, especially with some of the sources providing contradicting information. For example, Jiang et al. reported that even though a tendency for lower ADC values to coincide with pleural malignancy was observed, it was not statistically significant [4]. Additionally, the MRI’s potential to provide information on the tumor’s histopathology would have to be further investigated and developed, if it were to compete with the traditional diagnostic methods.

Outside of the pleural neoplasms’ detection, targeting the contrast enhancement areas or diffusion restriction areas in MRI could be useful for pleural biopsy guiding [78], in order to further increase its accuracy.

In 2000 Bonomo et al. wrote about a tendency to under-stage the MPM based on the imaging, which was of lesser importance in the case of tumors in the resectable T1–T3 stages, but highly relevant in the case of the unresectable T4-stage tumors [13]. The problem was also reported in 2016 during the 13th International Conference of the IMIG [112]. It had been observed that as a result of surgical procedures tumors were often found to be of a higher stage than the one that was established based solely on the imaging [112]. We found multiple reports suggesting that similarly to CT, MRI was unable to precisely recognize the T1a, T1b, and T2 disease stages because it could not accurately distinguish the parietal pleura’s involvement from visceral pleura’s involvement [13,74,79], and neither could it detect the invasion of the diaphragmatic muscle [13,74,79] or the pericardium [74,79]. However, it could be used to recognize the T3 and T4 disease stages through the detection of diaphragmatic and endothoracic fascia invasion, as well as the detection of single foci of chest wall invasion [13,74,79,104], in whose detecting it was better than the CT [13,104]. On the contrary, according to Mylene et al. [3] both CT and MRI were unable to detect microscopic disease in MPM, which made them a poor choice for detecting subtle transdiaphragmatic extension in locally advanced disease (T4 according to the TNM International Staging System for Diffuse MPM). Because of that, laparoscopy and peritoneal lavage were recommended in the case of the pre-operative patients, to better judge the tumor’s resectability [3]. In 2010, in a review paper, Helm et al. reported that some studies had shown high sensitivity (above 90%) of both MRI and CT in terms of resectability prediction, however with very low specificity (between 25% and 50%) [8]. The authors concluded that the MRI seemed to be an inferior staging tool in comparison to the pathological diagnosis [8].

MR has also been suggested as an alternative imaging tool to CT for the post-resection follow-up examinations of SFT patients [125] which would allow the radiation dosage received by them to be lowered.

Despite some promising results, further research with a larger sample size is required [88,107,151,155,156]. Such a project could be especially difficult to carry out due to the low prevalence of primary pleural tumors.

Additionally, to this point we lack information about the MRI’s use in patients with extremely rare pleural tumors which must be considered during the differential diagnostics of patients with pleural lesions. It seems that in a large number of the rare pleural tumor cases the MRI was applied for obtaining more detailed lesions’ descriptions; however, the first-line imaging methods were usually Roentgen or CT, which is most likely a reflection of the methods’ availability and the current pleural imaging recommendations. In the analyzed case reports regarding rare pleural tumors, we observed that although CT use was more common, the MRI has been used relatively often to obtain additional information and better judge the tumor’s extent prior to the use of more invasive diagnostic tools and treatment methods. This would support the information obtained by researchers in the case of more common pleural tumors, in which they often found that the MRI provided additional or more exact information on the tumor’s location, character, or extent, which made it an especially valuable method in more complicated cases.

An important limitation is the MRI’s ability to detect the pleural tumors’ metastases. It has been demonstrated that PET/CT could also be used for MPM staging and might be better than MRI for malignant lymph nodes’ detection and distant metastases’ identification [156]. According to an analysis by Zahid et al. [89] published in 2010, in which the authors discussed the contents of multiple research papers regarding the effective MPM staging, PET/CT was superior to FDG-PET, MRI, and CT in detecting and staging the MPM [89]. Hall et al. [102] found that DCE-MRI performed on MPM patients prior to chemotherapy could provide some prognostic information; however, they found the FDG-PET/CT results to be more significant [16]. In 2016 Botticella et al. wrote that the FDG-PET/CT should be used as a standard for N- and M-staging in MPM patients [114]. However, MRI could be used for high-quality T-staging which would increase the prognostic accuracy and promote more appropriate treatment selection [114]. Murphy et al. [87] stipulated that the combination of the FDG-PET/MRI could be an optimal tool to retrieve possibly exact staging data, which could lead to a better, more personalized patient approach. Although according to some research, the CT’s superior spatial resolution seemed to make it a better tool for the detection of small pleural and pulmonary nodules, in comparison to the MRI [87], the use of ultrashort free-breathing MRI sequences could provide a solution to that. It has been reported that the T2-gated PROPELLAR sequence delivered images without breathing artifacts, which improved its capacity for nodules’ detection [87]. Moreover, the free-breathing MRI sequences would be optimal for patients experiencing breathing difficulties, e.g., MPM patients [87]. An important downside of PET/CT is that it is based on glucose metabolism which is not specific to malignancies [151]. The enhancement can be a result of an infection or an inflammation which could lead to false-positive or uncertain results [120,151]. In addition, the talc pleurodesis in MPM patients could cause local inflammation and affect the FDG distribution, which could falsify the FDG-PET results [87]. Schaarschmidt et al. have reported that PET/MRI seemed to be a good alternative to PET/CT [156]. Furthermore, Coolen et al. found that DWI could correct the false-positive results obtained through PET/CT [151]. If these results are confirmed in larger studies, PET/MRI could be used as a lower radiation dosage alternative to PET/CT.

The respiratory motion may cause artifacts in the MRI, which could be avoided with the use of breath-holding, respiratory gating, and navigator techniques [8,13,14,16,102]. The occurrence of cardiac motion-related artifacts in the MRI could be minimized by cardiac gating and the use of ultra-short sequences [8,13,14,74].

Another issue is the selection of the most appropriate method for the images’ evaluation. In a retrospective analysis of the CE CT and MR images obtained from 29 patients, Pena et al. [15] found that the radiomics texture and shape analysis could aid physicians in image interpretation and improve the precision of the currently available methods. The researchers found that radiomics could improve the accuracy of some radiologists’ MR and CT images’ analysis, especially if the analyzing physicians were not specialized in thoracic radiology [15]. In a study on 19 MPM patients, Mehndiratta et al. [157] compared the DCE MRI grey-scale images and color-coded images—the images were rated by eight radiologists. The researchers found that the color-coded images had a better diagnostic value and a superior tumor vasculature display [157]. Tomšič et al. found that two models—ET (extended Tofts) and AATH (adiabatic approximation tissue homogeneity model)—were appropriate for predicting therapy response in MPM patients [22]. However, in another study Lee at el. compared the use of five tracer kinetic models (Tofts-Kety, ET, AATH, two compartment exchange model, and a distributed parameter model) for the DCE MRI’s analysis [63]. The researchers found that the two-compartment exchange model was optimal for the assessment of the MPM’s microvascular properties; however, the study only included 5 MPM patients and 10 NSCLC patients [63]. Moreover, Tomšič et al. observed that the significance of certain parameters changed not only depending on the used DCE analysis model but also depending on the utilized statistical analysis (univariate vs. multivariate) [107].

Additionally, some of the results could depend on the disease stage of the enrolled patients.

Further research should be based on inter-institutional cooperation and include a higher number of patients, possibly in all disease stages. The analytical models used for the analysis of the selected MRI sequences should be investigated in a comparative study. The differences in the parameters’ significance based on the analysis type and used analytical model should be considered.

## 6. Conclusions

Although we seem to be rather far away from replacing the histopathological examination with a method not requiring invasive sampling as a method of choice for the pleural tumor’s final diagnosis, the MRI could be used to improve the accuracy of the currently applied standard methods and should be considered as an alternative to CT for the pre-operative tumor’s assessment. MRI has been shown to have certain advantages over CT and when combined with PET it could also be more efficient than PET/CT. Due to the strong development of MRI within the last two decades, imaging possibilities have grown significantly. We are currently provided with a number of relatively new and promising MRI techniques and a variety of methods for their analysis, which must be researched further in the context of pleural imaging in order to select the most exact and robust options.

So far, the biggest issue seems to be a low number of pleural neoplasms which restricts the number of patients that could be enrolled into studies based in one or two clinical units, which coincides with a wide range of MRI techniques, analytical models and measured parameters that need to be researched. For that reason, the currently available studies focus on selected aspects of the MR’s applications in pleural imaging and are usually restricted in terms of the subjects’ selection—either by a low number of patients or by their disease stage. We are in dire need of larger studies that would recruit more patients in all disease stages, consider multiple MRI techniques (e.g., CE and DWI), utilize various analytical models (if such can be applied), and include all the parameters described by such models which have so far been shown to perhaps carry a diagnostic value. In order for such studies to happen, a well-coordinated multi-institutional cooperation would be required which could be especially hard to orchestrate due to the differences in the clinics’ equipment, staff experience, and funding possibilities. However, if carried out successfully such studies could be a way to identify the most efficient options for the pleural malignancies’ diagnosis.

## Figures and Tables

**Table 1:** The classification of the non-metastatic pleural neoplasms [20,21].

Mesothelial Tumours		Lymphoproliferative Disorders	Mesenchymal Tumours
Benign and Preinvasive Mesothelial Tumours	Mesothelioma		
Adenomatoid tumour	Localized mesothelioma	Primary effusion lymphoma	Epithelioid haemangioendothelioma
Differentiated papillary mesothelial tumour	Diffuse mesothelioma	Diffuse large B-cell lymphoma associated with chronic inflammation	Angiosarcoma
Mesothelioma in situ	Both have subtypes:Sarcomatoid mesothelioma		Synovial sarcoma
	Epithelioid mesothelioma		Solitary fibrous tumour
	Biphasic mesothelioma		Calcifying fibrous tumour
			Desmoplastic round cell tumour

**Table 2:** This table provides an overview of the pleural tumors’ presentation in various MRI sequences. DWI was not included.

Tumour Type	T1-Weighted MRI	Dynamic and DCE (Dynamic Contrast Enhanced) MRI	T2-Weighted MRI
MPM	Pleural thickening, nodules, masses—isointense/mildly hyperintense in relation to the chest wall muscle * [1,2,3,13,75,76,82,84,85,104]. Pleural effusions—low signal intensity [10,92].	Moderately enhanced signal after gadolinium administration [75,76]. Pleural thickening—diffusely enhanced [92].	Pleural thickening, nodules, masses—moderately hyperintense in relation to the chest wall muscle [1,2,3,13,75,76,77,84,85,104]. Unilateral pleural effusion—focal high signal intensity [2,9,82]. Pleural fluid—focal hyperintense areas [13]. Pleural effusions—high signal intensity [10,75,92].
Solitary fibrous tumour	Tumour—low/intermediate intensity due to the fibrous tissue’s presence [2,13,17,75,79,123,124].	After gadolinium—intense homogenous enhancement reflects the tumour’s vascularity [11,75,79,125].	Tumour—low/intermediate intensity due to the mature fibrous tissue’s presence [2,11,17,75,79,123,128]. Highly intense heterogenous signal—possibly a reflection of the tumour’s high cellularity [2,13,55] **. High signal intensity in areas of necrosis and myxoid degeneration [11,75,79,123]. Internal septations—low signal intensity [2]. Tumour may have a low intensity margin [75,79]. Malignant fibrosis—high signal intensity caused by increased vascularity, cellularity and edema [124].
Lipoma	High signal intensity [2,74,79,88]. Well-defined homogenous mass—hyperintense [17,74,79].		Well-defined homogenous mass—moderate signal intensity [17,74,79,88].
Liposarcoma	Heterogenous signal—a mixture of fat and soft tissue [2]. Low signal intensity (myxoid degeneration) [17,74,75,79].	Uneven enhancement [75].	High signal intensity (myxoid degeneration) [17,74,75,79].
PEL (primary effusion lymphoma)			Effusion -hyperintense signal [2,9]. Cystic/necrotic regions may occur after systemic therapy—high signal intensity [2]. Pleural thickening and nodules/masses may be observed.
Pleural lymphoma	Hypo or isointense in comparison to the chest wall muscle [126].	Contrast enhancement present in fat suppressed T1 MRI [126].	Hyperintense [126].
Hemangioma	Mass—high signal intensity [135].	Eccentric enhancement in the early-phase images and filling in in the delayed-phase scans [135].	Mass—high signal intensity [135].
Pleural Schwannoma	Tumour—hypo- to isointense in relation to muscle [143,144]. Split fat sign may be present [144].	After gadolinium—uneven signal enhancement [143].	Tumour—inhomogeneous areas with peripherally hyperintense and centrally hypointense structures [143]. Cystic degeneration with hyalinization—hyperintense (due to poor blood flow and degeneration) [143], may also be hypointense [144].
Pleural neurofibroma	Low-intensity signal [141].		Heterogenous high-intensity signal [141].
Primary pleuropulmonary synovial sarcoma	Tumour mass—heterogenous medium-intensity signal. Necrotic regions—hypointense. Hemorrhages—hyperintense [132].	Heterogenous enhancement in T1 [132].	Tumour mass—heterogenous medium-intensity signal. Necrotic regions—hyperintense. Hemorrhages—hypointense [132]. “Tripple sign” [131].
Leiomyoma	Isointense signal [129].	Heterogenous enhancement in T1 images [129].	Heterogenously highly intense signal [129].
Pleural low-grade fibromyxoid sarcoma	Hypo- or isointense to muscle. Myxoid edges or hemorrhagic effusion—mild hyperintensity [133].	Ring-shaped enhancement [133].	Heterogenous highly intense signal. Myxoid tissue—hyperintense. Fibrous tissue—hypointense [133].

* They can be enhanced through the intravenous administration of the gadolinium-based contrast material [1]. ** The source [75] reports a rather low-intensity heterogenous signal.

**Table 3:** This table contains a comparison of exudative and transudative pleural fluid whose MRI-based analysis could help identify a cause of the pleural effusion containing it.

	Exudate	Transudate
Diffusion in DWI	Low diffusion [74,75].	High diffusion [74,75].
Signal intensity in triple echo imaging	High signal intensity [74].	Low signal intensity [74].
Related conditions	Malignancy, infection, thromboembolic disease [75].	Increased hydrostatic pressure, decreased colloid osmotic pressure [75].
